# High-Risk Chronic Lymphocytic Leukemia Complicating the Course of Imatinib-Treated Chronic Myeloid Leukemia: Successful Disease Management With Dual Tyrosine Kinase Inhibition

**DOI:** 10.1155/2024/1813512

**Published:** 2024-11-15

**Authors:** Daniel James, Simone Green, Stefano Molica, David Allsup

**Affiliations:** ^1^Department of Haematology, Castle Hill Hospital, Hull University Teaching Hospital NHS Trust, Cottingham, UK; ^2^Centre for Biomedicine, Hull York Medical School, University of Hull, Hull, UK

**Keywords:** chronic lymphocytic leukemia, chronic myeloid leukemia, tyrosine kinase inhibitors

## Abstract

The coexistence of chronic myeloid leukemia (CML) and chronic lymphocytic leukemia (CLL) in the same patient is exceedingly rare, with only a few cases reported in the literature. Here, we report a patient with CML who, having achieved a major molecular response with imatinib, subsequently developed CLL, which necessitated the concomitant administration of ibrutinib.

## 1. Introduction

Chronic myeloid leukemia (CML) and chronic lymphocytic leukemia (CLL) are distinct haematological malignancies that arise from different haematopoietic lineages. CML is characterised by the chromosomal translocation *t*(9; 22) (q34.1; q11.2) and associated expression of the BCR-ABL1 tyrosine kinase [1]. Targeted therapy with tyrosine kinase inhibitors (TKIs) such as imatinib is the cornerstone of CML management, typically resulting in significant improvements in patient outcomes. CLL, in contrast, represents a clonal expansion of neoplastic B cells which coexpress the cell-surface antigens CD5, CD19, CD20 and CD23 with the expression of either *κ* or *λ* immunoglobulin light chains [2]. CLL is often managed with Bruton tyrosine kinase inhibitors (BTKi) such as ibrutinib and acalabrutinib or inhibitors of B-cell lymphoma-2 (BCL-2) such as venetoclax.

The incidence of CML ranges from 0.4–1.75 per 100,000 persons, and of CLL around 1.28 per 100,000 [3, 4]. Although not mutually exclusive, the coexistence of CML and CLL in the same individual is extremely rare and has been reported only sporadically in the literature [5–9]. There is therefore limited evidence on the concomitant treatment of CML and CLL with dual TKI and BTKi therapy.

Herein, we report a patient with CML who, having achieved a major molecular response (MMR) with imatinib, subsequently developed CLL, which necessitated the concomitant administration of ibrutinib without apparent toxicity. Written consent was obtained from the patient to publish this case report.

## 2. Case Presentation

**Figure 1:**
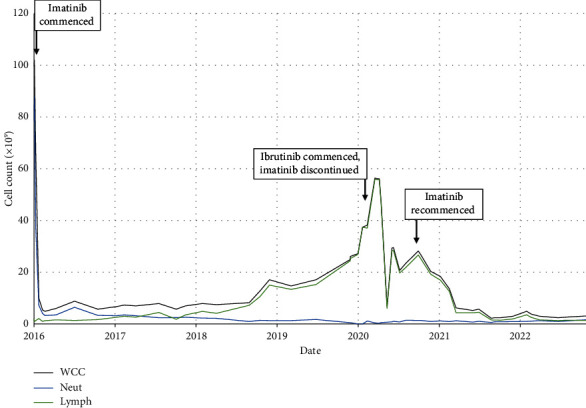
Cell counts throughout the study period.

**Figure 2:**
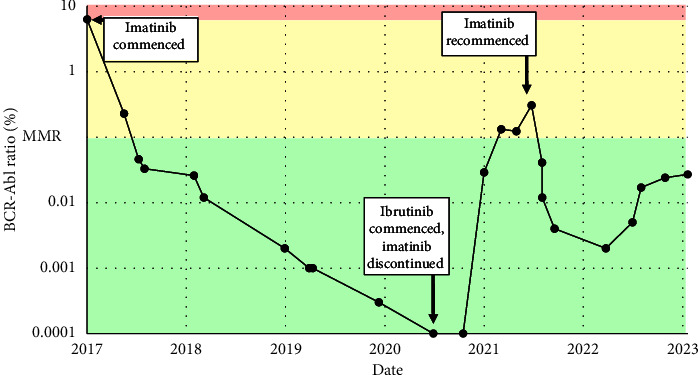
BCR-Abl ratio throughout the study period. MMR; major molecular response.

A 69-year-old male presented originally with increased fatigue and anorexia in the setting of extreme leukocytosis and a blood film appearance consistent with CML. His past medical history was notable for ischaemic heart disease which required the insertion of three coronary stents, glaucoma and colonic carcinoma managed with surgical resection without the administration of adjuvant chemotherapy. On examination, there was no palpable lymphadenopathy, but hepatosplenomegaly was noted, palpable 1 cm below the costal margins. At presentation, a bone marrow was performed which showed that less than 5% of leukocytes were CD19-positive B cells. Cytogenetic analysis on the bone marrow at the point of diagnosis of CML revealed *t*(9; 22) and 46, XY, *t*(9; 22) (q34; q11)[12). The patient's Sokal score was calculated as 0.95, and the Hasford score was 853.1, both of which conferred an intermediate disease risk [10, 11]. Treatment with imatinib was commenced at a dose of 400 mg once daily. The patient tolerated imatinib well with a complete haematological response at 3 months with normalisation of white cell count (Figure 1), and a subsequent MMR at 6 months with a BCR-ABL:ABL ratio < 0.1% (Figure 2).

The patient maintained an MMR over the subsequent 3 years on the same dose of imatinib. During this period, however, he had presented to his primary care physician with persistent dyspnoea at rest and on exertion following a lower respiratory tract infection. A routine chest X-ray revealed patchy consolidation of the lungs, and a follow-up computerised tomographic (CT) scan revealed ill-defined ground glass nodular opacities with a peribronchial distribution, in addition to widespread lymphadenopathy within the mediastinum, hilar regions, axillae and abdomen. The maximal para-aortic lymph node size was 3.5 × 2.2 cm. The patient had no change to his appetite, no weight loss, and no night sweats. The patient had a normal white cell count with no neutrophilia or lymphocytosis. The treating medical team considered that these changes were unrelated to the known CML and he was referred to a chest physician for further assessment. Assessments of antineutrophilic cytoplasmic antibody, angiotensin-converting enzyme, lactate dehydrogenase and creatine kinase were all negative or normal. A positron emission tomographic (PET) scan showed variable fluorodeoxyglucose (FDG) uptake, most intensely within the largest mesenteric and para-aortic lymph nodes (standard uptake value (SUV) maximum 3.7). Immunofixation revealed a small IgG kappa monoclonal band. No obvious cause for the abnormalities was identified, and the patient was monitored clinically without further investigation.

Two years in October 2020, following the prior CT and PET scan, the patient developed night sweats, weight loss and felt generally unwell. Assessment of the blood count revealed an hemoglobin of 88 g/L and a white cell count of 27 × 10^9^/L with a marked lymphocytosis. A bone marrow aspirate and biopsy revealed the marrow to be hypercellular with an extensive infiltrate of small lymphocytes with cell surface immunophenotype consistent with CLL, with flow cytometry showing the presence of neoplastic CD19+ CD20+ CD5+ CD23+ Kappa+ B lymphocytes. Fluorescent in situ hybridisation analysis revealed a chromosome 17p deletion with associated TP53 mutation, characterised by high throughput sequencing (HTS) as c.592G > T with a variant allele frequency of 0.751. HTS also identified a variant of unknown significance in TNFAIP3. Multiplex ligation dependent probe amplification revealed 12q24.33 (CHFR)^loss^ and 17p13.1 (TP53)^loss^. Assessment of the immunoglobulin heavy chain mutational profile was not performed.

Imatinib was subsequently discontinued to facilitate the delivery of CLL therapy, as the patient had achieved a sustained deep molecular response for more than 12 months at this point, and ibrutinib 420 mg once daily was commenced whilst imatinib was discontinued. The total white cell count gradually declined over the next 12 months and a complete haematological response as per International Workshop on CLL criteria was seen 2 years after the commencement of ibrutinib [2]. The BCR-ABL:ABL ratio rose from 0.000% to 0.132% after being off imatinib for 3 months.

Imatinib was therefore restarted at a dose of 100 mg once daily and increased to 300 mg once daily over the subsequent 3 months, taken alongside ibrutinib, resulting in the reattainment of MMR (Figure 2). The doses of both imatinib and ibrutinib have subsequently been slowly titrated, and the patient continues to simultaneously take both imatinib 300 mg once daily and ibrutinib 140 mg once daily for the concurrent management of both CML and CLL.

On his most recent assessment, the CLL was in a minimal residual disease positive complete remission with 0.25 × 10^9^ CLL-cells/L whilst there was a continued CML MMR with a BCR-ABL:ABL ratio of 0.029%. Whilst on dual imatinib/ibrutinib therapy the patient has had a persistent mild neutropenia (neutrophils 0.9–1.6 × 10^9^/L) unresponsive to G-CSF, corresponding to a CTCAE grade II [12] and a normocytic anemia (Hb 106–119 g/L). He has also developed a localised squamous cell carcinoma (SCC) in the neck, likely related to pre-existing scalp SCCs treated by excision, which is for treatment with the immunotherapeutic agent cemiplimab and concomitant radiotherapy.

## 3. Discussion

The case highlights a rare coexistence of CML and CLL managed with concomitant imatinib and ibrutinib, reported only sporadically in the literature [5–9]. Secondary malignancies are not uncommon in CML, although analyses are contradictory as to whether there is an increased risk in persons with CML as compared to the general population. More recent analyses of cohorts managed with TKI therapy do not describe any difference in the risk of solid cancers in persons with CML [13]. The CML Study IV did, however, find a significant increase in the risk of non-Hodgkin lymphoma in persons with CML [13]. It is known that imatinib interferes with the effector function of T-lymphocytes and disrupts the differentiation of peripheral haematopoietic progenitors, which may encourage the development of secondary haematological malignancies [14]. A recent case report described the development of CML in a person with CLL treated with ibrutinib who subsequently was treated with venetoclax, a BCL-2 inhibitor, with a clinical response [8]. A second report described the development of CML on a background of refractory CLL, managed to good effect with simultaneous imatinib and ibrutinib [9]. It is possible that the SCC in our patient is a result of dual TKI therapy; however, it is also known that nonhaematological malignancies are present with an increased incidence in CLL and indeed carries a higher morbidity and mortality than in the general population [15]. In addition the presence of both deletion 17p and somatic TP53 mutations in the CLL clone could indicate a constitutional susceptibility to malignancy, however we did not undertake constitutional genetic analysis in this case.

Our case also provides anecdotal evidence of the safety and efficacy for the co-administration of ibrutinib and imatinib. We initially discontinued imatinib when CLL was diagnosed to safely commence ibrutinib. We eventually restarted imatinib due to a rising BCR-ABL:ABL ratio, but fortunately found that the patient tolerated dual TKI therapy very well with only mild neutropenia and bruising. A lower dose of imatinib was initially prescribed due to its known activity as a potent inhibitor of CYP3A4, a characteristic shared with ibrutinib [16, 17].

It may be possible that due to the broad range of kinases targeted by both imatinib and ibrutinib, there may be a synergistic anti-tumour effect against the CML or CLL clones. Imatinib has been shown to induce apoptosis in BCR-ABL-negative CLL lymphocytes at clinically relevant concentrations, potentially through interaction with c-abl and the proapoptotic protein Par-4 [18].

The case presented in this report adds to the descriptions of the rare subset of patients with coexistent CML and CLL and demonstrates the tolerability and efficacy of imatinib and ibrutinib prescribed in conjunction. The case presented in this report adds to the descriptions of the rare subset of patients with coexistent CML and CLL, and demonstrates the tolerability and efficacy of imatinib and ibrutinib prescribed in conjunction. Further work to identify the optimum management of this subset of patients with the simultaneous administration of multiple TKIs is required.

## Data Availability

The data used to support the case report are included within the article.

## References

[1] Arber D. A., Orazi A., Hasserjian R (2016). The 2016 Revision to the World Health Organization Classification of Myeloid Neoplasms and Acute Leukemia. *Blood*.

[2] Hallek M., Cheson B. D., Catovsky D (2018). iwCLL Guidelines for Diagnosis, Indications for Treatment, Response Assessment, and Supportive Management of CLL. *Blood*.

[3] Lin Q., Mao L., Shao L (2020). Global, Regional and National Burden of Chronic Myeloid Leukemia, 1990–2017: A Systematic Analysis for the Global Burden of Disease Study 2017. *Frontiers Oncology*.

[4] Ou Y., Long Y., Ji L (2022). Trends in Disease Burden of Chronic Lymphocytic Leukemia at the Global, Regional, and National Levels From 1990 to 2019, and Projections Until 2030: A Population-Based Epidemiologic Study. *Frontiers Oncology*.

[5] Rahman K., George S., Mangal S., Mehta A. (2013). Simultaneous Occurrence of Chronic Myeloid Leukemia and Chronic Lymphocytic Leukemia: Report of an Unusual Case. *Indian Journal of Pathology & Microbiology*.

[6] Bhagavathi S., Borromeo V., Desai H., Crisan D. (2008). Case Report and Literature Review: A Rare Patient With Chronic Myeloid Leukemia and Chronic Lymphocytic Leukemia. *Annals of Clinical Laboratory Science*.

[7] D’Arena G., Gemei M., Luciano L (2012). Chronic Lymphocytic Leukemia After Chronic Myeloid Leukemia in the Same Patient: Two Different Genomic Events and a Common Treatment?. *Journal of Clinical Oncology*.

[8] Przespolewski E. R., Baron J., Kashef F (2023). Concomitant Venetoclax and Imatinib for Comanaging Chronic Lymphocytic Leukemia and Chronic Myeloid Leukemia: A Case Report. *Journal of the National Comprehensive Cancer Network*.

[9] Shea L. K., Mikhail F. M., Forero-Torres A., Davis R. S. (2017). Concomitant Imatinib and Ibrutinib in a Patient With Chronic Myelogenous Leukemia and Chronic Lymphocytic Leukemia. *Clinical Case Reports*.

[10] Sokal J. E., Cox E. B., Baccarani M (1984). Prognostic Discrimination in “Good-Risk” Chronic Granulocytic Leukemia. *Blood*.

[11] Hasford J., Pfirrmann M., Hehlmann R (1998). A New Prognostic Score for Survival of Patients With Chronic Myeloid Leukemia Treated With Interferon Alfa. Writing Committee for the Collaborative CML Prognostic Factors Project Group. *Journal of the National Cancer Institute*.

[12] *Common Terminology Criteria for Adverse Events (CTCAE) Version 5.0*.

[13] Miranda M. B., Lauseker M., Kraus M. P (2016). Secondary Malignancies in Chronic Myeloid Leukemia Patients After Imatinib-Based Treatment: Long-Term Observation in CML Study IV. *Leukemia*.

[14] Appel S., Balabanov S., Brümmendorf T. H., Brossart P. (2005). Effects of Imatinib on Normal Hematopoiesis and Immune Activation. *Stem Cells*.

[15] Lai M., Pampena R., Cornacchia L (2022). Cutaneous Squamous Cell Carcinoma in Patients With Chronic Lymphocytic Leukemia: A Systematic Review of the Literature. *International Journal of Dermatology*.

[16] Filppula A. M., Laitila J., Neuvonen P. J., Backman J. T. (2012). Potent Mechanism-Based Inhibition of CYP3A4 by Imatinib Explains its Liability to Interact With CYP3A4 Substrates. *British Journal of Pharmacology*.

[17] De Zwart L., Snoeys J., De Jong J., Sukbuntherng J., Mannaert E., Monshouwer M. (2016). Ibrutinib Dosing Strategies Based on Interaction Potential of CYP3A4 Perpetrators Using Physiologically Based Pharmacokinetic Modeling. *Clinical Pharmacology & Therapeutics*.

[18] Chow K. U., Nowak D., Hofmann W., Schneider B., Hofmann W. K. (2005). Imatinib Induces Apoptosis in CLL Lymphocytes With High Expression of Par-4. *Leukemia*.

